# Equity and access to maternal and child health services in Ghana a cross-sectional study

**DOI:** 10.1186/s12913-021-06872-9

**Published:** 2021-08-24

**Authors:** Samuel George Anarwat, Mubarik Salifu, Margaret Atosina Akuriba

**Affiliations:** 1 School of Public Health, Department of Health Services, Policy, Planning, Management and Economics (HSPPME), University for Development Studies, Tamale, Ghana; 2 School of Applied Economics and Management Sciences, Department of Economics, University for Development Studies, Tamale, Ghana

**Keywords:** Maternal and child health, Equity, Health service access and delivery, Concentration index, Risk ratios, Ghana, Universal health coverage

## Abstract

**Background:**

Inequities in the distribution of and access to maternal and child health care services is pervasive in Ghana. Understanding the drivers of inequity in maternal and child health (MCH) is important to achieving the universal health coverage component of the Sustainable Development Goals (SDGs) and poverty reduction in developing countries. However, there is increasing disparities in MCH services, especially in rural -urban, and income quintiles. The study aimed to examine the disparities in maternal and child health care services in Ghana for policy intervention.

**Methods:**

Data for this study was extracted from the nationally representative Ghana Statistical Service (GSS) Multiple Indicator Cluster Survey (MICS) round 4, 2011. Respondents of this survey were women of reproductive age 15–49 years with a sample size of 10,627 households. The models were estimated using multivariate regression analysis together with concentration index (CI) and risk ratio (RR) to assess the distribution of MCH indicator groups across the household wealth index.

**Results:**

The results show that women with secondary school level and above were more likely to receive family planning, prenatal care, and delivery by a skilled health professional than those without formal education. Mothers with low level of educational attainment were 87% more likely to have their first pregnancy before the age of 20 years, and 78% were more likely to have children with under-five mortality, and 45% more likely to have children who had diarrhoea. teenage pregnancy (CI = − 0.133, RR =0.679), prenatal care by skilled health worker (CI = − 0.124, RR =0.713) under five mortality, child underweight, reported diarrhoea, and suspected pneumonia, though not statistically significant, were more concentrated in the poorer than in the richer households, The RR between the top and bottom quintiles ranged from 0.77 for child underweight to 0.82 for child wasting.

**Conclusion:**

Geographic location, income status and formal education are key drivers of maternal and child health inequities in Ghana. Government can partner the private sector to implement health policies to address inequalities in MCH services through primary health care, and resource allocation skewed towards rural areas and the lower wealth quintile to bridge the inequality gaps and improve MCH outcomes. The government and the private sectors should prioritize female education, as that can improve maternal and child health.

## Background

Improved maternal and child health is imperative for the survival of every nation and global population. However, inequities in the distribution of and access to maternal and child health care services is pervasive in Ghana. Understanding the drivers of inequity in maternal and child health (MCH) is important to achieving the universal health coverage component of the Sustainable Development Goals (SDGs) and poverty reduction in Low – and Middle-Income Countries. The study aimed to examine inequities in maternal and child health care services among population groups especially, the upper and lower wealth quintiles on one hand and geographical (Rural-Urban) disparities in access to MCH on the other hand. The study contributes to filling in the gaps in literature and inadequacy of equity in MCH data in Low -and Middle-Income Countries (LMICs) for evidence-based MCH policy and programme implementation. Knowing the gaps and the determinant factors of inequities in MCH can aid in effective resource allocation and MCH programs implementation to improve health outcomes.

Ghana has embarked on several interventions and efforts aimed at preventing and reducing maternal and infant mortality. These interventions span enhancing utilisation of health care through the National Health Insurance Scheme (NHIS), Free Maternal Care programme, launched in 2007 for pregnant women to enhance their utilisation of delivery care services, Healthcare Network (CHN)-On-The-Go, Kangaroo Baby Care, Mobile Technology for Community Health (MOTECH), and Millennium Development Goal Acceleration Framework (MAF), and Expanded Program on Immunization, among others. It is expected that the NHIS will improve provision of basic health care services to persons resident in the country.

Inequities in the distribution of health care services is gaining global attention in public health [1]. Inequities in maternal and child health services are among the prominent reasons for high maternal and child morbidities and mortalities in Sub-Saharan Africa and the world at large [2]. Like other Sub-Saharan countries, Ghana experiences unequal distribution in maternal and child health care services [3]. Quality and equitably access to MCH is critical for the improvement of health outcomes and health status of women and children. However, inequitable spread of MCH services increases mortality in resource poor regions. Infant, child, and maternal mortality are highest in Northern Ghana, where poverty is rife and access to MCH services are woefully in short supply. The poor MCH services in Northern Ghana is worse than the economically endowed Southern Ghana (Greater Accra and Ashanti) regions. It is estimated that 1 in 27 infants in Ghana die before their first birthday, and 1 in 19 children die before age five [4].

In 2017, the Maternal Mortality Ratio was estimated at 310 per 100,000 deaths, with a lifetime risk of maternal death estimated at 1% of all women dying from maternal causes. A stunning 517 pregnant women die of maternal causes, annually in Ghana, for lack of quality maternal health care services, and inequitable distribution of MCH services [4]. Surprisingly, most of the maternal and infant mortalities are preventable [5]. Ghana’s key health status indicators on MCH, such as maternal mortality rate (MMR), infant mortality rate (IMR), family planning, and neonatal mortality rate (NMR) improved steadily over the MDG period. This achievement was praised by the international community as a remarkable success. Nonetheless, there were large inequalities in health coverage and strong overall performance marks significant disparities between income groups and regions [6]. Much is still left to be accomplished in the areas of equity in maternal and child health status among the lower wealth quintile of the population. The 2017/2018 Ghana Multiple Indicator Cluster Survey (MICS), round six report showed a reduction in infant mortality rate (IMR) from 49 per every 1000 to 41 children per 1000 live births, according to the Ghana Statistical Services data in 2018. This statistic is far from achieving the SDG Goal 3-Target 3.2 of “ending preventable deaths of new-borns and children under 5 years with all countries aiming to reduce neonatal mortality.

Low or no education of mothers also hampers their maternal and child health status and well-being. In Ghana low education of mothers is still a problem, considering that as many as 79 maternal deaths were associated with mothers with no education while 53 deaths for mothers with at least secondary education [7]. Inequality creates serious obstacles to achieving the Sustainable Development Goals (SDGs), especially, overcoming poverty, achieving universal health coverage and reduced inequalities by the year 2030 (SDG1, 3 and 10). The widening and persistent inequality in the distribution of social services including maternal and child health care is harmful to countries as well as individuals as agreed by policy makers [8]. Between 2016 and 2017, 82% of the wealth generated went to the richest 1% of the global population, while the poorest half saw no increase [6]. Over 800 women die globally every day from complications in pregnancy and childbirth due to the disparities in maternal and child health services utilization [7]. For every woman who dies, approximately 20 others suffer serious injuries, infections or disabilities and almost all maternal deaths (99%) occur in developing country regions [7]. According to the Population Reference Bureau [9] the maternal mortality rates (MMR) for Africa and West Africa are 490 per 100,000 live births and 674 per 100,000 live births, respectively. These statistics are worrying since no woman should die giving birth. The survival of mothers has become very important since saving them implies saving the lives of the more than one million children who are left motherless.

Research shows that educated women are more likely to start antenatal care (ANC) visits earlier than less educated women [10], and utilisation of delivery care depends largely on the women’s educational level [11]. Most maternal health studies in Ghana [12, 13], have focused mostly on the general population, and in some instances on some regions. Arthur (2012), for instance, identified wealth, age, education, number of children, transportation, and health insurance among women between 15 and 49 years to influence antenatal use in Ghana as contributory factors of maternal health care service utilisation. Other variables such as long travel distance and long waiting times affect the use of ANC within communities [14]. We present in the next few paragraphs, previous literature on maternal health, family planning, and child health in Ghana.

### Maternal health

Ghana has made good progress in recent years in many social development indicators including health. Maternal and child health status are determined by several variables such as the conditions of the place of residence, school environment, and work environment, which determines their health risks and outcomes. Environmental and social variables such as, health care and early health care seeking and treatment; educational attainment of households especially mothers, employment, social support and economic opportunities, family incomes, health insurance coverage, significantly influence maternal health care behaviors and health status [13]. These variables that influence maternal health equally affect pregnancy outcomes and child health status [15].

Evidence suggests high unmet need for family planning among unmarried adolescents while modern use of family planning methods is higher among married than unmarried adolescents [3]. Adolescent girls in rural areas and those among the poorest and less educated are at a higher risk of early childbearing in Ghana [3]. Ample evidence indicates that 14% of adolescent women aged 15–19 are mothers or pregnant with their first child [3].

### Family planning

Family planning has been an integral component of government of Ghana’s maternal health programs for decades. Family Planning is an important factor in the population management and national development outlined in many national development plans [16].

Family planning aims to assist couples and individuals of reproductive age to achieve their reproductive aspirations. Despite the high premium placed on family planning programs in Ghana, funding remains a daunting challenge. Family planning intake is highest (69%) among women between 15 and 19 years and lowest (33%) among women within 45–49 years. The demand for family planning is also highest (59%) among women in rural areas. Those women with at least primary or high school education use more family planning services and women in the middle three quintiles (60–61%) [17].

Although there is a huge progress in family planning services intake, there is still about 50% unmet need for family planning services in Ghana, especially among young women within the 15–19 years (51%) and lowest among women aged 45–59 (14%). Also women in rural dwelling have slightly higher (31%) unmet needs of family planning than their counterparts in urban areas (29%) [17].

Delivery by skilled health personnel is another key indicator of maternal health. There has been progress in this indicator, about 68% of all births in the last 2 years preceding the MICS survey round 6 were delivered by skilled personnel. Education plays an important role in deliveries by skilled health personnel. Educated woman were more likely to have assisted delivered by a skilled health personnel. Assisted delivery by skilled health personnel for mothers with no formal education constituted only 44% of all deliveries compared to 95% for women with secondary or higher education. Also, poor women were less likely to deliver using skilled personnel (39%), compared to rich women (98%). Despite the progress made in delivery by skill personnel deliveries at home is still highly significant as 1 in 3 births take place at home without a skilled health personnel [18]. This needs to be addressed to reduce preventable maternal mortality which might emanate from complications or blood loss.

Promoting and ensuring deliveries in health facilities can reduce the health risks to both the mother and the baby. Proper medical attention and hygienic conditions during delivery can also reduce the risks of complications and infection that can cause morbidity and mortality to either the mother or the baby [18].

### Child health

The health of children is a global concern. Over the years, many countries and institutions have worked towards improving the health of children to reduce infant mortality. Despite the significant investments and improvement in child health in the past few decades, many children still lose their lives to diseases before their 5th birthday globally, and inequity in health is still a huge challenge [19]. Technical and medical solutions such as disease control and medical care of illnesses that cause the most deaths in children are critical in making fundamental improvements in health equity.

Diarrhoea and pneumonia have been the most frequent childhood illnesses and causes of attendance at health facilities in low-income and middle-income countries [20]. These diseases have been regarded as the “biggest child killers” in the last century [21]. In 2011 for instance, diarrhoea and pneumonia caused about 700,000 and 1, 300,000 global deaths respectively in children under 5 years [20]. The plan was necessitated by some alarming statistics that pneumonia and diarrhoea together accounted for 30% of all childhood deaths [22]. The prevalence rate of diarrhoea in children under 5 years in Ghana is reported to be 13% in the 2011MICS, with Oral Rehydration Treatment (ORT) being higher in urban areas (64%) than in rural areas (56%) [18]. Childhood diarrhoea in Ghana is also said to show inequities that are to the disadvantage of the poorest [23]. Thus, cases recorded in urban areas or in relatively rich homes are properly managed compared to those in rural and/or poor homes. In 2011, the incidence of suspected pneumonia in children under 5 years in Ghana was 3% [18]. The main intervention in treating pneumonia is antibiotics. Of the 3% suspected pneumonia cases reported in Ghana, 41% of them were taken to an appropriate health provider and 56% received antibiotics. Children in rural areas and/or poor homes are disadvantaged in terms of care seeking behaviour [18].

WHO estimates that at least 10 million deaths were prevented between 2010 and 2015 globally due to vaccinations; and many lives were protected from suffering and disability associated with diseases such as pneumonia, diarrhoea, whooping cough, measles, and polio [21]. However, in Ghana many children still suffer from nutrition deficiency illnesses and other preventable childhood diseases. For instance, children under 5 years suffered from stunting (18.8%) underweight (11.0%) and wasting (4.7%) [18]. There are also significant variations in stunting, underweight, and wasting across wealth quintiles and geographic regions [24]. An estimated 19% of Ghanaian children are chronically malnourished, with stunting less than two standard deviations (SD) below the national average, and 5% are stunted (below − 3 SD). This represents a 17% decrease (improvement) since the MICS in 2011 and a 47% decrease (improvement) since the DHS in 2008 [22].

Stunting becomes more common as children get older, peaking at 28% among children aged 24–35 months. Stunting affects a significantly higher percentage of males (20%) than females (17%), and stunting is more prevalent in rural areas (22%) than in urban areas (15%). Stunting rates vary by area, ranging from 10% in Greater Accra to 33% in the Northern region. Education and income are inversely linked to stunting. For example, 25% of children in the lowest two wealth quintiles are stunted, while only 9% of children in the highest quintile are stunted [22].

## Methods

### Data sources

Data for this study was extracted from the 2011 Ghana Multiple Indicator Cluster Survey, round four (MICS4 (19). The Ghana Multiple Cluster Indicator survey (MICS) is a nationally representative survey which contains valuable data on the condition of children, women, and men in Ghana. Unlike the previous MICS, the Ghana MICS4 2011 included three “malaria biomarkers,” such as anaemia testing, malaria testing using rapid diagnostic tests (RDTs), and thick blood smear samples prepared on microscope slides.

### Study settings

The data were collected in all regions of Ghana namely, Greater Accra, Central, Western, Volta, Eastern, Volta, Ashanti, Brong Ahafo, Northern, Eastern, Upper East and Upper West regions. The estimated population of Ghana, as of July 2021 was estimated at 31,754,995 people based on Worldometer elaboration of the latest United Nations data. The population density is estimated at 137 per Km^2^ (354 people per mi^2^), and the Urban Population was 56.7% (17,625,567 people in 2020). The total land area is 227,540 Km2 (87,854 sq. miles). https://www.worldometers.info/world-population/ghana-population

### Sample design

The MICS 2011 used a cross-sectional sample design to collect data on multiple indicators about children, women, and men, nationwide, stratified into urban and rural areas and the 10 geographical regions of Ghana.

### Questionnaires/instruments

The Ghana MICS4, 2011, used four different sets of questionnaires in the survey:1) Household questionnaire which collected information on usual residents, the household, and the dwelling, 2) Women’s questionnaire data on women aged 15–49 years, 3) Under-5 questionnaire administered to mothers or caretakers for all children under 5 living in the household, 4) Men’s questionnaire administered in each third household to all men aged 15–59 years.

The household questionnaire contained Household listing form, Education, Water and Sanitation, Household Characteristics, Insecticide Treated Nets, Indoor Residual Spraying, Child Labor, Child Discipline, Handwashing, and Salt Iodization.

The Women questionnaire included Women’s Background, Access to Mass Media and Use of Information/Communication Technology, Child Mortality, Desire for Last Birth, Maternal and Newborn Health, Post-natal Health Checks, Illness Symptoms, Contraception, Unmet Need, Female Genital Mutilation/Cutting, Behaviour Change Communication on Malaria, Attitudes Towards Domestic Violence, Marriage/Union, Sexual Behaviour, HIV/AIDS, and National Health Insurance.

The Children Under-Five Questionnaire (administered to mothers or caretakers of children under- 5 years of age) included, Age, Birth Registration, Early Childhood, Development, Breastfeeding, Diet Diversity, Care of Illness, Malaria, Immunization, National Health Insurance, Anthropometry, Anaemia, and Malaria Testing.

The Men Questionnaire (administered to men aged 15–59 years living in each third Household) contained Men’s Background, Access to Mass media and use of Information/Communication Technology, Marriage/Union, Attitude Towards Contraception, Behaviour Change Communication on Malaria, Attitudes Towards Domestic Violence, Sexual Behaviour, HIV/AIDS National, Health Insurance.

The questionnaires were pre-tested in two districts: Ga West district in Greater Accra region and Akwapim South district in Eastern region and finalized. For the study, we used the composite household questionnaire.

### Sampling method

The sampling frame used for the sampling was the 2000 Ghana Population and Housing Census data. The urban and rural areas of each region served as the key sampling strata. Two stage sampling method was used. In each stratum, the established population census enumeration areas were then systematically selected using probability proportional to size. There is no self-weighting because some of the regions namely Central, Northern, Upper East and Upper West regions were over-sampled. However, sample weights are used in reporting national level results.

The sample consists of women within the ages of 15–49 years with a live birth in the last 5 years preceding the survey. Of the 12,150 households sampled, 10,963 women aged 15–49 years were interviewed, with a response rate of 97%. Children under the age of 5 years constituted 7626. Responses were obtained from their mothers or caregivers with a response rate of 99%. The male survey involved 3511 men aged 15–59 years with a response rate of 95%. The questionnaire had questions on demographic indicators, health status, illness and visits to a doctor, health behaviour such as smoking, drinking alcohol, physical activity, and eating habits. We obtained our variables from the composite household survey, cleaned for missing values, and analyzed.

### Inclusion and exclusion criteria

Only the composite household questionnaires with questions on MCH were included for the analysis based on our variables. All the other questionnaires were not included in the analysis which did not have data on the key variables of interest were not included.

### Data analysis

#### Measurement of inequities

We measured inequities in maternal and child health outcomes and access to health care interventions by three steps: i) Identification of the health outcome or intervention whose distribution is to be measured; ii) classification of the population into different strata by a selected equity stratifier; and iii) measuring the degree of inequality [23]. Finally, we tried to understand the drivers of these inequities in MCH utilization. The variables of interest, maternal and child health outcomes and interventions are listed in Table 1. In the Multiple Indicator Cluster Survey, the socio-economic stratifier used is household wealth, which is derived from the household ownership of assets such as television, car etc. and dwelling characteristics such as flooring material and source of drinking water. In this study, we have used wealth quintiles that are provided in the MICS 4 report [18]. Each asset was assigned a weight (factor score) generated through principal components analysis, and the resulting asset scores were standardised in relation to a normal distribution with a mean of zero and standard deviation of one. Each household was then assigned a score for each asset, and the scores were summed for each household; individuals were ranked according to the total score of the household in which they resided. The sample was then divided into quintiles from one (lowest) to five (highest). A single asset index was developed for the whole sample; separate indices were not prepared for the urban and rural populations [24].

**Table 1:** Concentration index and ratio of richest to poorest quintile or decile for maternal health indicator of maternal and child health

MCH indicator	CI	RR (quintile5: quintile1)^**a**^	RR (decile 10: decile 1)^**b**^
1. **Maternal health**			
**Outcome indicator**			
I. Teenage pregnancy	−0.133***	0.679**	1.064
**Coverage indicator**			
II. Family planning	0.076***	1.408***	1.448**
III. Prenatal care by skilled health worker	−0.124***	0.713***	0.526***
IV. Delivery care by skilled health worker	0.026	1.195	1.031
V. Delivery care in health facility	0.287***	3.104	1.161***

*CI* Concentration index, *RR* Risk ratio

^a^ Quintile 5 is the richest; quintile 1 is the poorest

^b^ Decile 10 is the richest; decile 1 is the poorest

*P < 0.1; **P < 0.05; ***P < 0.01

To date, various measures have been used in the measurement of inequities in health and health care. However, of the available measures only the slope index of inequality (SII), the relative index of inequality (RII) and the concentration index (CI) have been commonly used giving their desirable characteristics: (i) they reflect the socio-economic dimension of health inequalities; (ii) they reflect the experience of the entire population rather than only two groups such as wealth quintiles one and five and (iii) they are sensitive to changes in the distribution of the population across socio-economic groups [25].

#### The concentration index

In this study, inequities in maternal and child health are measured using the concentration index. The concentration index is defined with reference to the concentration curve, which is used to identify whether socioeconomic inequality in some health sector variable exists and whether it is more pronounced at one point in time than another or in one country than another. But a concentration curve does not give a measure of the magnitude of inequality that can be compared conveniently across many time periods, countries, regions, or whatever may be chosen for comparison. However, the concentration index quantifies the degree of socioeconomic related inequality in a health variable [26, 27]. The CI has been used, for example, to measure and to compare the degree of socioeconomic-related inequality in child mortality [28], child malnutrition [29], health subsidies [30], and health care utilization [31].

Formally, the concentration index is defined as:

1 $$ C=1-2\underset{0}{\overset{1}{\int }}{L}_h(p) dp. $$

The index is bounded between − 1 and 1. For a discrete living standards variable, it can be written as:

2 $$ \boldsymbol{C}=\frac{\mathbf{2}}{{\boldsymbol{N}}_{\boldsymbol{\mu}}}\sum \limits_{\boldsymbol{i}=\mathbf{1}}^{\boldsymbol{n}}{\boldsymbol{h}}_{\boldsymbol{i}}{\boldsymbol{r}}_{\boldsymbol{i}}-\mathbf{1}-\frac{\mathbf{1}}{\boldsymbol{N}} $$

where ***h***_***i***_ is the health sector variable, *μ* is its mean, and ***r***_***i***_ ***= i***, *N* is the fractional rank of individual *i* in the living standards distribution, with *i*  = 1 for the poorest and *i*  = *N* for the richest. For computation, a more convenient formula for the concentration index defines it in terms of the covariance between the health variable and the fractional rank in the living standards distribution [26, 32, 33].

3 $$ \boldsymbol{C}=\frac{\mathbf{2}}{\boldsymbol{\mu}}\boldsymbol{\operatorname{cov}}\ \left(\boldsymbol{h},\boldsymbol{r}\right) $$

The sign of the concentration index indicates the direction of any relationship between the health variable and position in the living standards distribution, and its magnitude reflects both the strength of the relationship and the degree of variability in the health variable.

#### Equity stratifies and measures

Health equity is the absence of unjust, avoidable differences in health care access, quality, or outcomes. Measuring health inequalities allows us to identify differences that can be acted on and can be used to measure progress toward achieving health equity. Disaggregating health indicators using equity stratifiers can identify inequalities between subpopulations. An equity stratifier refers to a characteristic such as a demographic, social, economic, racial, or geographic descriptor that can identify population subgroups for the purpose of measuring differences in health and health care that may be considered unfair or unjust [34].

To assess wealth, the study used selected assets and durables in a sample household, because asset ownership tends to fluctuate less than individual income or expenditure. The assets considered in houses were permanent floors, roofs, or walls; flush or pour-flush toilets; transportation - including bicycles, motorcycles, cars or trucks; and electrical equipment, including radios, televisions, line or mobile telephones, refrigerators and computers. Households with these assets were considered richer than those without. The study also used principal component analysis of all household samples to generate a wealth index for each household and use this as an equity stratifier. This was done by measuring final use of goods and services, and money payments to obtain them and measures asset ownership, housing and/or access to services. This was then used to construct asset indices using methods such as principal component analysis. Using the concentration standard method, the study summarized the distribution of each MCH indicator over a gradient of the wealth index by a concentration index (CI) and a concentration curve (CC). The CI, which ranges from − 1.0 to + 1.0, captures the extent to which health outcomes and service use were concentrated among different population groups: the richest and the poorest. A CI of zero means an equal distribution of a particular indicator throughout the economic gradients. A negative CI indicates a concentration among those who are poorer (i.e., the CC lies above the equality line of 45 degrees), and a positive CI reflects a concentration among those who are richer (i.e. the CC lies below the equality line).

The study also compared the prevalence of health outcomes and the coverage of the MCH interventions between the richest and the poorest subgroups using a risk ratio (RR). All households were ranked according to their wealth indices, which was divided equally into quintile [5] and decile [9] subgroups. Only the top (richest) and bottom (poorest) quintiles and deciles were selected for the RR calculation, to demonstrate any disparity between rich and poor urban and rural domiciles and educated and uneducated.

Multiple regression models were then used to assess the drivers of inequity in the MCH outcomes using CI and RR as dependent variables. Data was analysed using STATA 13 statistical software.

## Results

### Disparities among the poor and the rich in MCH

Tables 1, 2, 3 and 4, summarizes equity measures for all MCH indicators in terms of CI and RR between the richest and poorest quintiles and deciles.

**Table 2:** Concentration index and ratio of richest to poorest quintile or decile for child health indicator of maternal and child health

MCH indicator	CI	RR (quintile5: quintile1)	RR (decile 10: decile 1)
2. **Child health Outcome indicator**			
VI. Low birth weight	−0.021	0.861	0.815
VII. Under five mortalities	−0.247***	0.426***	0.350***
**Child malnourishment**			
Underweight	−0.055***	0.772**	0.837
Stunting	−0.029	0.738**	0.740**
Wasting	−0.003	0.822**	0.791**
**Child illness**			
Diarrhoea	−0.145***	0.392***	0.386***
Suspected pneumonia	−0.002	1.002	0.927
**Coverage indicator**			
VIII. ORS/ORT for diarrhoea	−0.221***	0.344***	0.485
IX. Appropriate provider for pneumonia	0.111***	1.704***	1.715*
X. **Child immunization**			
BCG	0.060**	1.598***	1.658***
MMR	0.054**	1.583***	1.589***
DPT	0.064**	1.640***	1.684***
Yellow fever	0.069***	1.673***	1.682***

*BCG* Bacille Calmette–Guerin, *CI* Concentration index, *DPT* Diphtheria, pertussis (whooping cough) and tetanus, *MMR* Measles, mumps and rubella, *ORS/ORT* Oral rehydration salts/oral rehydration therapy, *RR* Risk ratio

^a^ Quintile 5 is the richest; quintile 1 is the poorest

^b^ Decile 10 is the richest; decile 1 is the poorest

*P < 0.1; **P < 0.05; ***P < 0.01

**Table 3:** Prevalence and risk ratio with respect to urban–rural areas and educational attainment for maternal health indicator of MCH

MCH indicator	Average prevalence	Urban Vs. Rural		Higher Educ^a^. Vs. Lower Educ.	
	(%)	RR	*p*-value	RR	*p*-value
**Maternal health**					
**Outcome indicator**					
XI. Teenage pregnancy	7.93	0.731***	0.000	1.129***	0.000
**Coverage indicator**					
XII. Family planning	24.80	1.029	0.549	1.419***	0.000
XIII. Prenatal care by skilled health worker	26.02	0.701*** 0.000		1.457*** 0.000	
XIV. Delivery care by skilled health worker	15.72	0.983	0.797	1.304***	0.000
XV. Delivery care in a public health facility	13.52	1.001 0.992		1.365* 0.050	
XVI. Delivery Care in a private health facility	1.53	1.129	0.555	1.474***	0.000

^a^ Higher education refers to schooling beyond the secondary (High School) level

*P < 0.1; **P < 0.05; ***P < 0.01

**Table 4:** Prevalence and risk ratio with respect to urban–rural areas and educational attainment for child health indicator of MCH

MCH indicator	Average prevalence	Urban Vs. Rural		Higher Educ^a^. Vs. Lower Educ.	
	(%)	RR	*p*-value	RR	*p*-value
**4.Child health**					
**Outcome indicator**					
Low birth weight	16.73	1.001	0.994	0.894***	0.000
Under five mortality	24.85	0.671***	0.000	0.224***	0.000
Underweight	16.13	0.911	0.262	0.849***	0.000
Stunting	16.56	0.976	0.756	0.860***	0.000
Wasting	16.76	0.902**	0.043	0.875**	0.049
Child Illness: Diarrhoea	14.77	0.729***	0.001	0.552***	0.000
Child Illness: Suspected pneumonia	21.92	1.006	0.929	1.056	0.507
**Coverage indicator**					
Child Treatment: ORS/ORT for diarrhoea	2.83	0.860	0.522	0.776	0.360
Child Treatment: Appropriate provider for pneumonia	7.61	1.331**	0.014	1.602***	0.000
Child Immunization: BCG	15.11	1.465***	0.000	1.568***	0.000
Child Immunization: MMR	14.37	1.518***	0.000	1.574***	0.000
Child Immunization: DPT	13.35	1.525***	0.000	1.662***	0.000
Child Immunization: Yellow Fever	13.06	1.556***	0.000	1.631***	0.000

*BCG* Bacille Calmette–Guerin, *CI* Concentration index, *DPT* Diphtheria, pertussis (whooping cough) and tetanus, *MMR* Measles, mumps, and rubella, *ORS/ORT* Oral rehydration salts/oral rehydration therapy

^a^ Higher education refers to schooling beyond the secondary level

*P < 0.1; **P < 0.05; ***P < 0.01

#### Economic disparities in health outcomes

We discovered substantial differences between income classes and geographic areas, suggesting that household wealth has a significant impact on child survival and that the poor have a higher risk of child mortality. Teenage pregnancy (CI = − 0.133, RR =0.679), Prenatal care by skilled health worker (CI = − 0.124, RR =0.713) (See Table 1); low birth weight (CI = − 0.021) (though not statistically significant), under five mortality (CI = − 0.247, RR = 0.426); all child malnourishment indicators: underweight, stunting and wasting (CI =, − 0.055, − 0,029, − 0.003, RR =0.772, 0.738, 0.822) respectively, and child disease all showed economic inequalities in MCH (See Table 2). The poorer subgroups were more likely to have negative health effects (as shown by the negative CIs in Tables 1 and 2). The poor had the highest concentration, which was statistically important for child underweight. The CI for stunting and wasting in children was negative. In terms of magnitude of concentration, teenage pregnancy was ranked third among the poor. Children under the age of 5 years old with suspected pneumonia and diarrhoea were also more prevalent among the poor, though not statistically significant (Table 2). The RRs were compatible with the computed CI when comparing MCH outcomes between the top and bottom income quintiles. Teenage pregnancy, under-five mortality, underweight children, and confirmed diarrhoea and suspected pneumonia were all more common in poorer households than in wealthier ones. The RR between the upper and bottom quintiles ranged from 0.77 for underweight children to 0.82 for wasting children (Table 2). Low birth weight was less clearly associated with economic disparities. The negative CI represented the fact that it was concentrated in relatively poor families, but it had no statistical significance.

#### Economic disparities in service coverage

The results indicate that the primary MCH interventions were spread more evenly across economic strata than the health outcomes (Table 2). Prenatal service by a professional health worker was statistically significant and concentrated among the poor, while delivery care in a health facility was also statistically significant and concentrated among the wealthy. The magnitude of the CI, on the other hand, was high, and the RR between the wealthiest and poorest groups was not close to one, suggesting a large disparity between the wealthy and the poor. The poorest quintile had the highest coverage of oral rehydration salts/oral rehydration therapy for diarrhoea. The wealthiest quintile and decile, on the other hand, had the highest coverage of adequate health-care services for suspected pneumonia. Table 2 shows that the CIs of the four vaccine types for childhood immunization coverage ranged from 0.054 to 0.069, and the CI for family planning (at 0.076) is all statistically different from zero. As a result, since all the indicators were clustered around the richest quintiles and deciles, there were inequalities in service coverage for these indicators. The RR for these indicators had mixed results (Tables 1 and 2); both had more than one for comparisons between the first and fifth quintiles, as well as between the first and tenth deciles.

#### Geographic inequity of MCH

Health outcome prevalence’s were as follows: teenage pregnancy among all mothers, 7.93%; low birth weight, 16.73%; and child stunting, 16.56% (Table 4). Service coverage was less equitable; for example, on average, less than 26% of respondents had prenatal care delivered either by skilled health workers or in health facilities, and less than 15% of children had received all vaccinations.

Tables 3 and 4 also summarizes the urban–rural and educational disparities in MCH, as reflected by the RR. The four vaccines (BCG, MMR, DPT and yellow fever) and the coverage indicator appropriate provider for pneumonia were all concentrated in the urban areas than rural areas (thus, by 47, 52, 53 and 56% respectively) while low birth, under five mortality, underweight, stunting, wasting, child illness (diarrhoea and suspected pneumonia) and coverage indicator ORS/ORT for diarrhoea were more concentrated in rural than in urban areas. The most profound health gap was under-five mortality, which was 33% more prevalent in rural than in urban areas.

The urban–rural gap for MCH service coverage was quite large. For instance, women living in rural areas were 30% less likely than those in urban areas to receive prenatal and delivery care from a skilled health worker. Although family planning was concentrated in the urban areas than rural, this was not statistically significant. Again, teenagers in urban areas were 73% less risky in getting expose to teenage issues compared to their counterparts in the rural areas. Also, there was a sharp gap between urban and rural women usage of print/electronic media and technology, thus women in urban areas were 4.9–78% more likely to use newspapers, radio, television, computer, and internet than their counterparts in the rural areas.

#### Educational inequity and MCH disparity

Mothers’ or caregivers’ formal schooling is a significant determinant of MCH inequity. Our findings show that more educated mothers or caregivers did better on all outcome indicators. The disparity was most noticeable when it came to teenage pregnancy. Women with less than a secondary school education were 87% more likely than those with a secondary school education to have their first pregnancy before the age of 20. Mothers or caregivers with no formal education were more likely (78% and 45%, respectively) to have under-five mortality and children with diarrhoea than those with a secondary education (Table 3).

Women with education beyond secondary school were 30–46% more likely than those without any formal education to receive family planning, maternal care, and delivery by a professional health worker or in a health facility. Higher educational attainment was also associated with a consistent improvement in maternal care coverage, with a large difference (RR: 1.304–1.457; *P* < 0.01). Surprisingly, children born to mothers or cared for by someone with a post-secondary education were 57–66% more likely than those who were not in this subgroup to receive all forms of vaccination before the age of 1 year.

## Discussion

The study analysed data from the Ghana Statistical Service’s (GSS) Multiple Indicator Cluster Survey (MICS 4, 2011) to unearth inequities in maternal and child health services delivery and access in Ghana. The results can inform policy planning to improve maternal and child health services, especially in the disadvantage areas and populations subgroups**.**

In 2016, Ghana’s MMR was 319 per 100,000 live births [32]. This demonstrates that MDG 5 [5], which aimed to reduce maternal mortality by three-quarters (190 deaths per 100,000 live births) by 2015, was not met. This means that if Ghana is to meet the Sustainable Development Goal (SDG) three [3], it will need to make more thoughtful and pragmatic efforts. Addressing and closing the disparities between rich and poor, educated, and uneducated, and social gradients in general are important first steps toward improving the well-being of mothers, infants, and children. Mothers’ and children’s health and socioeconomic well-being will have an impact on future generations’ health and make predicting future public health challenges for families, societies, and the health-care system easier. Much has been accomplished in terms of necessary child immunization, but ANC still has much room for improvement [22]. The sharp drop in ANC threatens to undo the country’s advances in maternal health care. With regional, location, mothers’ education, and wealth quintile inequalities, ANC coverage has dropped from 93.08% in 2012 to 81.3% in 2017 [33].

Major differences in MCH care measures were discovered in the literature across many geographical areas, maternal, economic, and socio-demographic factors in many developing countries. What is unknown are the factors that contribute to inequities in MCH service access and distribution.

Government health expenditure has a less progressive effect on inequality in Ghana than in many other nations, accounting for just one-third of the reduction in Gini Co-efficient [4]. Again, the government’s annual allocation to the health sector has fluctuated and remains below the Abuja Declaration target of 15% (ISSER, 2018), implying that less money is going into Maternal and Child Health services, further widening the country’s health disparities.

Assessing inequalities’ drivers is critical for making evidence-based decisions and allocating scarce public resources to those who are most in need. The existence of health-care inequities that disadvantage the poor, rural dwellers, and women with low education makes achieving the relevant health-related SDG targets difficult. This study sends a strong policy message: universal access to health care is critical to achieving the objective of health equity or reducing inequalities between sub-groups such as the poor and the wealthy, rural and urban dwellers, and those who have completed secondary school and those who have not completed secondary school.

In Ghana, child immunization was given to all mothers or caregivers regardless of their economic status. Surprisingly, immunization coverage was marginally higher in urban areas and among children whose mothers or caregivers had completed secondary school than in rural areas and among those who had not completed secondary school. These results indicate that urban areas have higher service coverage than rural areas.

Government’s low commitment to investment in health infrastructure and expansion of health insurance coverage over the past decades can explain Ghana’s relatively unequal distribution of MCH service coverage – between the wealthy and the poor, urban and rural populations [7]. Over the last few decades, the regional reach of district hospitals and sub-district health centers has tended to increase in favor of the urban and wealthy. District health systems, which include hospitals and health centers, are leading the way in offering a wide variety of curative, preventative, and health-promotion programs, including MCH.

In Ghana, there is still some difference in child health results between the rich and the poor, as well as between urban and rural areas. The country’s CIs for diarrhoea, malnutrition, underweight, and stunting, for example, are equivalent to the MICS for developing countries.

One of the most significant social determinants of health inequity is education. The education disparities for measures of teenage pregnancy and child malnutrition were far greater than the urban–rural differential, according to this report. As the mother’s or caregiver’s formal education level increased, the prevalence of adolescent pregnancy and infant malnutrition decreased. Teenage pregnancy was found to be much less common among those who had completed high school. This and other studies [12] show that maternal education is an important component of MCH policies.

In Ghana, access to MCH services is inequitable in the form of universal health coverage. Inequity in health outcomes is a problem, and social determinants (such as poverty, maternal education, and other structural social inequities) are significant, even though they are often beyond the health sector’s mandate. In this study, 16.13% of children were underweight, 16.56% were stunted, and 16.76% of children were wasting. Wasting in children suggests acute malnutrition, whereas stunting indicates chronic malnutrition, which is usually caused by long-term poverty in the home. Policies should tackle inequity from birth; for example, they should address problems including low birth weight, teen pregnancy, and infant malnutrition, for which the World Health Organization recommends multi-sectoral interventions. Inequity at birth has long-term consequences; undernutrition, for example, is linked to a loss of human capital (i.e., the skills and knowledge that enable people to work and thus produce economic value).

### Limitation of the study

The data for the study is somehow old and could have potential policy gaps. Many factors might have changed within the period 2011 to 2021. However, current literature on MCH has been incorporated into the paper to account for the limitations. Though the MICS4 data is somewhat old, the analysis is relevant for comparing the results with future studies that uses current MICS data. Also, the study would have benefited from in-depth qualitative understanding of the inequalities in MCH which was not conducted. Further qualitative research is needed to complement and enhance the understanding of the drivers of inequalities in MCH in Ghana.

## Conclusion

Major challenges remain in inequity in health outcomes, particularly in the areas of child mortality, teenage pregnancy, child malnourishment. The gaps between rich and poor and between urban and rural areas reveal a similar pattern. Mothers’ education is the main determinant of health inequity. Key policy leverage and multi-sectoral actions are needed to close these gaps. Inequities in most of the maternal and child health interventions in Ghana are widespread among sub-groups (rich-poor, urban-rural and high educated-less educated) to the detriment of poor, rural dwellers, and less-educated. Thus, tackling inequalities in resource allocation through primary health care services is key to fighting the extreme geographical and wealth inequality in Ghana. For instance, upping maternal and child health education and promotion, and improving the quality of primary health care clinics in the most disadvantage rural districts resource allocation using equity formulas can improve quality and equity of maternal and child health care in Ghana. Further research, especially qualitative research is needed to unravel the social dimensions of the inequalities and the congruent factors responsible for the MCH inequalities.

## Data Availability

The data that support the findings of this study are available from Ghana Statistical Service, but restrictions apply to the availability of these data, which were used under license for the current study, and so are not publicly available. Data are however available from Mubarik Salifu, *(our author who managed the data)* upon reasonable request and with permission of Ghana Statistical Service.
